# Respiratory tract infections among French Hajj pilgrims from 2014 to 2017

**DOI:** 10.1038/s41598-019-54370-0

**Published:** 2019-11-28

**Authors:** Van-Thuan Hoang, Saliha Ali-Salem, Khadidja Belhouchat, Mohammed Meftah, Doudou Sow, Thi-Loi Dao, Tran Duc Anh Ly, Tassadit Drali, Laetitia Ninove, Saber Yezli, Badriah Alotaibi, Didier Raoult, Philippe Parola, Vincent Pommier de Santi, Philippe Gautret

**Affiliations:** 1 Aix Marseille Univ, IRD, AP-HM, SSA, VITROME, Marseille, France; 2 IHU-Méditerranée Infection, Marseille, France; 3 Thai Binh University of Medicine and Pharmacy, Thai Binh, Viet Nam; 4 Service de Parasitologie-Mycologie, Faculté de médecine, Université Cheikh Anta Diop, Dakar, Senegal; 5 Unité des Virus Émergents (UVE: Aix-Marseille Univ – IRD 190 – Inserm 1207 – IHU Méditerranée Infection), Marseille, France; 6 The Global Centre for Mass Gatherings Medicine, Ministry of Health, Riyadh, Saudi Arabia; 7 Aix Marseille Univ, MEPHI, Marseille, France; 8 French Military Center for Epidemiology and Public Health, Marseille, France

**Keywords:** Risk factors, Molecular biology

## Abstract

Respiratory tract infections (RTIs) are common among Hajj pilgrims, but risk factors for RTIs and respiratory pathogen acquisition during the Hajj are not clearly identified. Based on previous studies, most frequent pathogens acquired by Hajj pilgrims were investigated: rhinovirus, human coronaviruses, influenza viruses, *Streptococcus pneumoniae*, *Staphylococcus aureus, Klebsiella pneumoniae* and *Haemophilus influenzae*. 485 pilgrims were included. 82.1% presented with RTIs. Respiratory chronic diseases were associated with cough, Influenza-like illness (ILI) and the acquisition of *H. influenzae*. Vaccination against invasive pneumococcal diseases (IPD) and influenza was associated with a decrease in the acquisition of *S. pneumoniae* and prevalence of ILI (aRR = 0.53, 95%CI [0.39–0.73] and aRR = 0.69, 95%CI [0.52–0.92] respectively). Individuals carrying rhinovirus and *H. influenzae-S. pneumoniae* together were respectively twice and five times more likely to have respiratory symptoms. Individual with *H. influenzae-K. pneumoniae* carriage were twice (p = 0.04) as likely to develop a cough. The use of disposable handkerchiefs was associated with a decrease in the acquisition of *S. aureus* (aRR = 0.75, 95%CI [0.57–0.97]). Results could be used to identify pilgrims at increased risk of RTIs and acquisition of respiratory pathogens. Results also confirm the effectiveness of influenza and IPD vaccinations in reducing ILI symptoms and acquisition of *S. pneumoniae* carriage respectively.

## Introduction

The Hajj is one of the largest annual religious mass gatherings in the world. Each year, Saudi Arabia attracts over 2 million pilgrims from over 180 countries, including about 2,000 from Marseille, France^1^. The Hajj presents major challenges in public health and infection control as the crowding conditions favor the acquisition, dissemination and transmission of pathogenic microorganisms^2^. Respiratory tract infections (RTIs) are particularly frequent during the pilgrimage and are responsible for most causes of hospitalization, with community-acquired pneumonia being a major cause of serious illness among pilgrims^3^. Many studies have been conducted among Hajj pilgrims over the last decade, demonstrating the high prevalence of respiratory symptoms and the frequent acquisition of respiratory pathogens^3–7^. The viruses most commonly acquired after the Hajj are human rhinovirus (HRV), human coronaviruses (HCoV) and influenza A virus (IAV). The most frequently acquired respiratory bacteria are *Streptococcus pneumoniae (S. pneumoniae)*, *Staphylococcus aureus (S. aureus)* and *Haemophilus influenzae (H. influenzae)*^3^. However, the etiology of RTIs at the Hajj is likely multifactorial and complex. The potential effects of vaccination against influenza and pneumococcus^8,9^, of non-pharmaceutical preventive measures including face-mask use and hand hygiene practice^10–12^ have been investigated, mostly based on clinical criteria, but results of studies are contradictory.

So far, to our knowledge, risk factors for pathogen acquisition during the Hajj are not clearly identified. Relationship between respiratory symptoms and carriage of respiratory pathogens at the Hajj also remain poorly understood making it difficult distinguishing between infection and colonization. We conducted this study to identify risk factors for respiratory symptoms and respiratory pathogens carriage at the Hajj, including socio-demographics, vaccination against influenza and invasive pneumococcal diseases (IPD) and adherence to non-pharmaceutical preventive measures using data collected from 2014 to 2017. We also evaluated the relationship between pathogen carriage, including multiple carriage and respiratory symptoms.

## Results

### Characteristics of study participants

The study enrolled 485 pilgrims, 96.5% of whom filled both the pre- and post-travel questionnaires. The study population had a median age of 61.5 years (interquartile = (52 – 68 years), min = 21, max = 96 years) and a male:female ratio of 1:1.3 (Table 1). The majority (88.4%) of the pilgrims were from North Africa. 66.3% had an indication for vaccination against IPD according to the French recommendation at the time of inclusion^13–20^. Diabetes (28.6%) and hypertension (29.5%) were the most common comorbidities (Table 1).

**Table 1:** Characteristics of the study population, Hajj pilgrims 2014-2017 (N = 485).

Variables		n	%
Pilgrimage year	2014	98	20.2
	2015	119	24.6
	2016	117	24.1
	2017	151	31.1
Gender	Male	212	43.7
	Female	273	56.3
Age*	Median	61.5	
	Interquartile	52–68	
	Min - max	21–96	
Age ≥ 60 years*		269	56.0
Place of birth	France	40	8.5
	North Africa	419	88.4
	Sub-Saharan Africa	13	2.7
	Others	2	0.4
Comorbidities*	Diabetes mellitus	136	28.6
	Hypertension	140	29.5
	Chronic respiratory disease	56	11.8
	Chronic heart disease	32	6.7
	Chronic kidney disease	5	1.1
	Immunodefiency	3	0.6
Indication for vaccination against IPD^1^		315	66.3
BMI^2^	Normal	130	26.8
	Underweight	3	0.6
	Overweight	220	45.4
	Obesity	132	27.2

^1^Indication for vaccination against invasive pneumococcal diseases: Age superior or equal to 60 years, diabetes mellitus, chronic respiratory disease, chronic heart disease, chronic kidney disease and immunodeficiency, (n = 475, 10 missing data).

^2^Body mass index. Normal weight: BMI: 18.5 – 24.9, Underweight: BMI <18.5, Overweight: BMI: 25.0 – 29.9, Obesity: BMI ≥30.

^*^n = 480, 5 mission data.

With regard to preventive measures, 96 (20.2%) pilgrims reported having been vaccinated against pneumococcal (PCV-13) in the last 5 years, representing 30.5% of pilgrims with an indication for IPD. 26.7% (127/466) were vaccinated against influenza before their travel or in the past year. Two hundred sixty-one (56.0%) pilgrims reported using face masks during the Hajj. Also, 42.1% (196/466), 50.4% (235/466) and 73.6% (343/466) pilgrims declared washing their hands during the Hajj more often than usual, using hand gel, and using disposable handkerchiefs during their stay in Saudi Arabia, respectively.

### Clinical features

**Figure 1:**
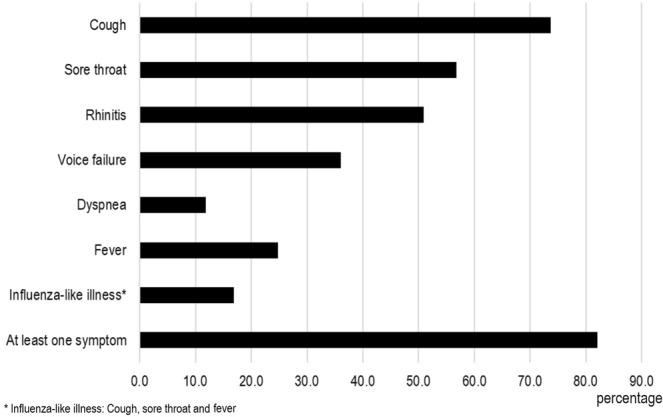
Prevalence of respiratory symptoms among pilgrims during the Hajj 2014–2017.

Figure 1 shows the prevalence of respiratory symptoms among pilgrims during the Hajj. More than 80% of the pilgrims presented at least one respiratory symptoms, cough, sore throat and rhinitis being the most frequent. Influenza-like illness (ILI) was present in 16.9% of pilgrims. None suffered from pneumonia or other IPD, and only one was hospitalized during the 2017 Hajj season. Time between arrival in Saudi Arabia and onset of symptoms was 9.5 ± 4.8 days (min = 0, max = 22 days).

### Detection of respiratory pathogens by qPCR

Pre- and post-Hajj specimens were collected from 456 (94.0%) and 451 (93.0%) participants, respectively. Furthermore, 32.6% (125/384) ill pilgrims were sampled at onset of respiratory symptoms. At total of 433 (89.3%) pilgrims had paired samples.

The prevalence of respiratory pathogens carriage increased after a participation in Hajj, compared to pre-Hajj status, and differences were significant for all bacterial pathogens and all viruses with the exception of HCoV-HKU1 (Table 2). Overall, 33.0% of pilgrims acquired at least one respiratory virus, notably RHV (26.9%), HCoV (8.0%) and influenza viruses (3.7%). A similar proportion (35.3%) acquired at least one respiratory bacterium after the Hajj, mainly *H. influenzae* (31.7%), *Klebsiella pneumoniae (K. pneumoniae)* (20.9%), *S. pneumoniae* (17.9%) and *S. aureus* (14.0%). Overall, viral-bacterial acquisition proportion in returned Hajjes was 26.9%, the most common being *H. influenzae*-virus dual carriage (Table 2). Among the 125 pilgrims sampled at symptom onset, a high prevalence of HRV (47/125, 37.6%), *S. aureus* (35/125, 28.0%) and *H. influenzae* (49/125, 39.2%) carriage was observed.

**Table 2:** Acquisition of respiratory pathogens during the Hajj.

Pathogens	Pre-Hajj		Per-Hajj		Post-Hajj		Acquisition		p*
	n = 456	%	n = 125	%	n = 451	%	n	%	
**Viruses**									
At least one virus	20	4.4	54	43.2	147	32.6	147	33.9	<10^−4^
All influenza viruses	0	0	6	4.8	11	2.4	16	3.7	<10^−4^
Influenza A	0	0	5	4.0	8	1.8	12	2.8	5.10^−4^
Influenza B	0	0	1	0.8	3	0.7	4	0.9	0.05
Rhinovirus	18	4.0	47	37.6	117	25.9	120	27.7	<10^−4^
All coronaviruses	2	0.4	17	13.6	29	6.4	36	8.3	<10^−4^
Coronavirus 229E	2	0.4	11	8.8	20	4.4	27	6.2	<10^−4^
Coronavirus HKU1	0	0	1	0.8	1	0.2	1	0.2	0.31
Coronavirus NL63	0	0	3	2.4	5	1.1	6	1.4	0.01
Coronavirus OC43	0	0	4	3.2	3	0.7	6	1.4	0.01
**Bacteria**									
At least one bacterium	217	47.6	78	62.4	344	76.3	159	36.7	<10^−4^
*S. aureus*	68	14.9	35	28.0	97	21.5	63	14.5	3.10^−3^
*S. pneumoniae*	18	4.0	5	4.0	89	19.7	80	18.5	<10^−4^
*H. influenzae*	127	27.9	49	39.2	232	51.4	144	33.3	<10^−4^
*K. pneumoniae*	58	12.7	15	12.0	115	25.5	94	21.7	<10^−4^
**Bacteria co**-**infections**									
*H. influenzae*-*S. pneumoniae*	9	2.0	2	1.6	49	10.9	47	10.9	<10^−4^
*H. influenzae*-*K. pneumoniae*	30	6.6	6	4.8	63	14.0	62	14.3	<10^−4^
*H. influenzae*-*S. aureus*	8	1.8	14	11.2	47	10.4	52	12.0	<10^−4^
*S. pneumoniae*-*K. pneumoniae*	4	0.9	1	0.8	23	5.1	24	5.5	2.10^−4^
*S. pneumoniae*-*S. aureus*	1	0.2	2	1.6	21	4.7	22	5.1	<10^−4^
*K. pneumoniae*-*S. aureus*	6	1.3	4	3.2	21	4.7	23	5.3	3.10^−4^
**Bacteria**-**virus co**-**infections**									
At least one virus-bacteria combinaison	6	1.32	29	23.2	113	25.1	120	27.7	<10^−4^
*S. pneumoniae*-virus	1	0.2	2	1.6	37	8.2	37	8.5	<10^−4^
*K. pneumoniae*-virus	1	0.2	8	6.4	34	7.5	41	9.5	<10^−4^
*S. aureus*-virus	2	0.4	13	10.4	36	8.0	39	9.0	<10^−4^
*H. influenzae*-*virus*	4	0.9	16	12.8	76	16.9	83	19.3	<10^−4^

^*^p value: pre-Hajj versus per-Hajj and/or post-Hajj, McNemar’s Test.

To compare between pre- and per-Hajj specimens, 122 ill pilgrims have had paired samples. The acquisition proportion of HRV, *S. aureus* and *H. influenzae* was 39/122 (32.0%), 14/122 (11.5%) and 43/122 (35.3%) respectively (data not show).

### Risk factors for respiratory symptoms

The Supplementary Table 1 and Table 3 show univariate and multivariate analyses results for factors associated with respiratory symptoms during the Hajj. Reporting of at least one respiratory symptom was twice as frequent and five times more frequent in rhinovirus carriers (adjusted relative risk (aRR): 1.98, 95%CI [1.03–3.78]) or *H. influenzae-S. pneumoniae* carriers, respectively (aRR: 4.75, 95%CI [1.17–19.35]) (Table 3). Coughing was twice as frequent in pilgrims suffering from chronic respiratory disease and among those carrying *H. influenzae-K. pneumoniae* together (aRR: 1.98, 95%CI [1.03–3.78]). Pilgrims who coughed were also more likely to use disposable handkerchiefs. Finally, ILI was more frequent in females, in pilgrims with chronic respiratory disease and among those carrying *S. aureus* or an association of virus and bacteria. In addition, pilgrims suffering ILI were more likely to use face mask. Influenza virus was not significantly associated with ILI. No significant association was observed between symptoms and vaccination against IPD. However, influenza vaccination was associated with a decrease in the prevalence of ILI (aRR: 0.69, 95%CI [0.52–0.92]).

**Table 3:** Risk factor for respiratory symptoms during the Hajj (multivariate analysis).

Variables	Cough		ILI^†^		At least one symptom	
	aRR [95%CI]	p	aRR [95%CI]	p	aRR [95%CI]	p
Male gender	—		0.75 [0.58–0.96]	0.02	—	
Chronic respiratory disease	2.24 [1.04–4.82]	0.04	1.47 [1.01–2.16]	0.05	—	
**Prevention measures**						
Mask	—		1.42 [1.10–1.82]	0.007	—	
Disposable handkerchiefs	1.71[1.17–2.50]	0.006	—		—	
Vaccination against influenza	—		0.69 [0.52–0.92]	0.012	—	
**Pathogens carriage during the Hajj**						
*Human rhinovirus*	—		—		2.03 [1.14–3.61]	0.02
*S. aureus*	—		1.34 [1.01–1.82]	0.044	—	
*H. influenzae – K. pneumoniae*	1.98 [1.03–3.78]	0.04			—	
*H. influenzae – S. pneumoniae*	—		—		4.75 [1.17–19.35]	0.03
At least one virus-bacteria combinaison	—		1.33 [1.01–1.74]	0.04	—	

^†^ILI: influenza-like illness.

*aRR: adjusted relative risk, CI: confidence interval, p: p value*.

We found no significant association between persistence of respiratory symptoms at return and pathogen carriage with the exception of *H. influenzae* carriage in association with viral carriage (aRR: 1.65, 95%CI [1.07–2.53], p = 0.02).

### Risk factors for respiratory pathogens acquisition among pilgrims

The Supplementary Tables 2 and 3 show the univariate risk factors analysis for respiratory virus and bacteria acquisition, respectively. In multivariate analysis (Table 4), male gender was associated with decreased acquisition of HCoV and *K. pneumoniae* (aRR: 0.76, 95%CI [0.58–0.99]). Older age (≥60 years) was associated with an increased acquisition of influenza viruses, HCoV and *S. pneumoniae* (aRR: 1.39, 95%CI [1.01–1.93], aRR: 1.53, 95%CI [1.16–2.01] and aRR: 1.28, 95%CI [1.01–1.63] respectively). Chronic respiratory disease was associated with increased acquisition of *H. influenzae* (aRR: 1.69, 95%CI [1.14–2.50]). No effect of vaccination against influenza was found. By contrast, vaccination against IPD was associated with a decrease in the acquisition of *S. pneumoniae* (aRR: 0.53, 95%CI [0.39–0.73]). Of note, IPD vaccination did not significantly influenced *S. pneumoniae* carriage at baseline (in pre-Hajj samples) (RR: 0.47, 95%CI [0.11–1.99], p = 0.29). Acquisition of HRV was higher among pilgrims who reported using facemasks (aRR = 1.30, 95%CI [1.03–1.65]). The use of disposable handkerchiefs has been associated with a decrease in the acquisition of *S. aureus* (aRR: 0.75, 95%CI [0.57–0.97]). Hand hygiene and the use of a disinfectant gel do not have a significant effect on the acquisition of pathogens.

**Table 4:** Risk factor for acquisition of respiratory pathogens during the Hajj (multivariate analysis).

Variables	Influenza viruses	Human rhinovirus	Human coronaviruses	*S. aureus*	*S. pneumoniae*	*H. influenzae*	*K. pneumoniae*
	aRR [95%CI] p	aRR [95%CI] p	aRR [95%CI] p	aRR [95%CI] p	aRR [95%CI] p	aRR [95%CI] p	aRR [95%CI] p
**Socio-demographics characteristic**							
Male gender			0.76 [0.58 – 0.99] 0.05				0.78 [0.62 – 0.98] 0.04
Age ≥60 years	1.39 [1.01 – 1.93] 0.04		1.53 [1.16 – 2.01] 0.003		1.28 [1.01 – 1.63] 0.05		
Chronic respiratory disease						1.69 [1.14 – 2.50] 0.01	
**Preventive measures**							
Vaccination against IPD					0.53 [0.39 – 0.73] < 0.0001		
Mask		1.30 [1.03 – 1.65] 0.03					
Handkerchief				0.75 [0.57 – 0.97] 0.03			

*aRR: adjusted relative risk, CI: confidence interval, p: p value, IPD: invasive pneumococcal diseases*.

## Discussion

Our results confirm that respiratory infections were very common among French Hajj pilgrims, with more than 80% of them reporting at least one symptom during the 2014–2017 Hajj seasons. Our result is in line with the previous results obtained from cohort studies conducted among French pilgrims^21^ and from a large cohort study enrolling pilgrims from 13 different countries^22^. We also document the significant acquisition of almost all viral and bacterial pathogens included in our survey, following participation to the Hajj, as reported previously^3,23^.

We found that male gender was independently associated with a decreased risk for ILI and for the acquisition of coronaviruses and *K. pneumoniae*. We have no explanation for this unexpected observation. Older age (≥60 years) was associated with the acquisition of influenza viruses, HCoV and *S. pneumoniae*. Although older age was not correlated with the increase in the proportion of respiratory symptoms in our study, our results support the current French recommendations that indicate influenza vaccination for all pilgrims and IPD vaccination for pilgrims aged ≥60 years old (or with chronic conditions)^24,25^. Furthermore, we showed that influenza vaccination was significantly associated with a lower prevalence of ILI in our survey. Similarly, in a meta-analysis, Alfelali *et al*. have shown that, influenza vaccine decreases the prevalence of ILI^26^. We also demonstrate here that vaccination against IPD with a decrease in the acquisition of *S. pneumoniae*, but had no effect on respiratory symptoms. A protective effect of pneumococcal vaccination against *S. pneumoniae* post-Hajj carriage was observed in only one out of 5 studies so far^9^. However, all studies showing no effect of pneumococcal vaccination were conducted on small groups of pilgrims (ranging 55 to 107 individuals), while in the two studies showing a protective effect, the number of pilgrims was much higher (1178 and 468) which may indicate that small surveys lacked statistical power. Finally, as expected, patients suffering from chronic respiratory diseases were more likely to suffer from cough and ILI but also to acquire *H. influenzae*. Our results confirm the need for influenza and IPD vaccination among the identified pilgrim populations at risk of RTIs and acquisition of respiratory pathogens. Vaccination rates in our cohort were clearly sub-optimal: 26.7% of pilgrims were vaccinated against influenza and 30.5% against IPD among those with an indication.

With regard to non-pharmaceutical preventive measures, the use of masks, especially in crowded areas, frequent hand washing with water and soap or disinfectant, especially after coughing and sneezing, and the use of disposable handkerchiefs are recommended by the Saudi Ministry of Health to Hajj pilgrims^27^. In a meta-analysis including 13 surveys of Hajj pilgrims, significant protection of face masks was found against RITs, but the end points varied considerably^12^. We found that there was a higher prevalence of ILI and rhinovirus acquisition among pilgrims who reported wearing face masks. Similarly, a higher prevalence of cough was observed among pilgrims who reported using disposable handkerchiefs. It is likely that such results indicate the higher willingness of symptomatic pilgrims to wear a face mask and use disposable handkerchiefs, with the aim of avoiding spreading diseases. In addition, the use of disposable handkerchiefs appears to be correlated with a decrease in the acquisition of *S. aureus* carriage, which may reflect a better elimination of organisms by cleaning the nasopharynx. We found that increased hand hygiene was not associated with reduced respiratory pathogens acquisition or a lower prevalence of respiratory symptoms. In a recent review paper, it was shown that while hand hygiene using non-alcoholic products was generally well accepted by Hajj pilgrims, there was no conclusive evidence of its effectiveness, which is consistent with our results^11^.

Relationship between respiratory symptoms and carriage of respiratory pathogens at the Hajj are unclear. Because of the high frequency of respiratory symptoms, the distinction between infection and colonization is difficult to assess. Furthermore, asymptomatic carriage of potential pathogens is also observed among Hajj pilgrims^28^. Nevertheless, in the final model of multivariate analysis, the acquisition of *S. aureus* was associated with ILI and the acquisition of rhinovirus was associated with respiratory symptoms. Many studies showed that *S. aureus* and rhinovirus were among the predominant pathogens isolated from the Hajj pilgrims suffering RTIs^9,11,12,21,23–32^. Most cases of infections due to HRV are benign, self-limited cold-like illnesses. Nevertheless, HRV is also responsible for severe pneumonia in the elderly and immunocompromised patients, as well as exacerbations of chronic obstructive pulmonary disease and asthma^33^. HRV spreads mostly via direct contact or contact with a fomite, with inoculation to the eye or nose from fingertips^34^. The human-to-human transmission of rhinovirus among pilgrims may have been favored by the crowded conditions at pilgrim accommodations or during performing the Hajj rites.

*S. aureus* is also often part of the human microbiome in the anterior nares^35^. However, persistent *S. aureus* nasal carriage is associated with secondary staphylococcal respiratory infection, predisposing to invasive disease, especially in the case of detection of IAV^36–38^. To date, few studies addressed virus-bacteria carriage in relation with RTIs at the Hajj^4,28^. In our study, overall carrying virus-bacteria was associated with ILI. Dual *H. influenzae-K. pneumoniae* carriage was associated with twice the risk of coughing and *H. influenzae-S. pneumoniae* carriage with 5 times the risk for respiratory symptoms. Further works aiming at better understanding the role of overall carrying virus and bacteria in the pathogenesis of the RTIs at the Hajj are needed.

Our study had some limitations. The study was conducted among French pilgrims only and cannot be generalized to all pilgrims. Observance with individual preventive measures was self-reported and frequency of changing face mask and exact quantification of hand hygiene practice were not assessed. qPCR used to detect respiratory pathogens does not distinguish between dead and living micro-organisms. *S. pneumoniae* serotypes were not investigated. *Moraxella catarrhalis* that was recently shown to be relatively frequently acquired by Hajj pilgrims^39^ was not included in our study. Finally, we did not to be any long-term follow-up to determine what infections might have had delayed presentation after return from the pilgrimage. Nevertheless, our study included a large samples size of pilgrims spanning four Hajj seasons giving it a stronger statistical power. A number of risk factors have been identified to recognize pilgrims at increased risk of RTIs and of acquisition of most common respiratory pathogens encountered in this setting. Also, the study confirmed the effectiveness of vaccination against influenza in reducing ILI symptoms and that of vaccination against IPD in reducing acquisition of *S. pneumoniae*. Given the limitations of the current study, the use of face mask and a reinforced hand hygiene should still be recommended for Hajj pilgrims until large-scale controlled studies are conducted to truly assess the effectiveness of these measures in the context of Hajj. The use of disposable handkerchief is, de facto, highly frequent among ill pilgrims and at least allows decreasing the acquisition of *S. aureus*.

## Methods

### Participants and study design

A convenience sample of pilgrims participating in the Hajj from 2014 to 2017 was surveyed. Potential adult participants were recruited at a private specialized travel agency from Marseille, France, organizing journeys to Mecca, Saudi Arabia and invited to participate in the study. They were included and followed-up by a medical bilingual (Arabic and French) doctor who traveled with the group. Upon inclusion before departing from France, the pilgrims were interviewed using a standardized pre-Hajj questionnaire that collected items about demographic characteristics (age, sex, place of birth), immunization status (vaccination against influenza and IPD) and medical history (chronic medical conditions, immunodeficiency, body mass index (BMI)). Pilgrims were considered to have been vaccinated immunized against influenza when they had been vaccinated within the last year, but before 10 days of the date of travel. Pilgrims were considered immune to IPD when they had been vaccinated with the 13-valent conjugate pneumococcal vaccine (PCV-13) in the past 5 years^13–20^. Clinical events occurring during the travel (type of respiratory symptoms, date of onset, duration of symptoms, antibiotic treatment provided) and persistence of respiratory symptoms at return were collected by a medical doctor using a post-Hajj questionnaire at 2 days before the pilgrims’ return to France. The information on compliance with face masks use as well as hand washing, use of hand gel disinfectant and disposable handkerchiefs was documented. Ill pilgrims, who spontaneously consulted the accompanying medical doctor at the time of onset during the Hajj, underwent a complementary nasopharyngeal swab (per-Hajj specimens). ILI was defined as the presence of cough, sore throat and subjective fever^40^. Based on the WHO classification, underweight was define as a BMI below 18.5, normal with a BMI from 18.5 to less than 25, overweight with a BMI greater ≥25 and obesity with a BMI greater ≥30^41^.

The protocol was approved by the Aix-Marseille University institutional review board (July 23^rd^, 2013; reference no. 2013-A00961-44). The study was performed according to the good clinical practices recommended by the Declaration of Helsinki and its amendments. All participants provided an informed written consent.

### Respiratory specimen

Nasopharyngeal swabs were obtained from each pilgrim, transferred to Sigma-Virocult^®^ medium and stored at −80 °C until processing. Pre-Hajj and post-Hajj nasopharyngeal swabs were systematically collected at enrollment, prior traveling to Saudi Arabia and two days prior returning to France, respectively. In addition, nasopharyngeal swabs were collected at symptom onset when possible.

#### Respiratory specimens

DNA and RNA were extracted from the respiratory samples using the EZ1 Advanced XL (Qiagen, Hilden, German) with the Virus Mini Kit v2.0 (Qiagen) according to the manufacturer’s recommendations. Quantitative real-time PCRs were conducted using a C1000 Touch™ Thermal Cycle (Bio-Rad, Hercules, CA, USA). Negative control (PCR mix) and positive control (DNA from bacterial strain or RNA from viral strain) were included in each run. Positive results of bacteria or virus amplification were defined as those with a cycle threshold (CT) value ≤35.

#### Identification of respiratory virus

One-step duplex quantitative RT-PCR amplifications of HCoV/HPIV-R Gene Kit (REF: 71–045, Biomérieux, Marcy l’Etoile, France) was used to detect HCoV and human para-influenza viruses according to the manufacturer’s recommendations. One-step simplex real-time quantitative RT-PCR amplifications were performed using the Multiplex RNA Virus Master Kit (Roche Diagnostics, France) for HRV, IAV, influenza B and internal controls MS2 phage^42^.

#### Identification of respiratory bacteria

Real-time PCR amplifications were carried out using the LightCycler^®^ 480 Probes Master kit (Roche diagnostics, France) according to the recommendations of the manufacturer. The SDD gene of *H. influenzae*, *nucA* gene of *S. aureus*, *phoE* gene of *Klebsiella pneumoniae*, *lytA CDC* gene of *S. pneumoniae*, were amplified with internal DNA extraction controls TISS, as previously described^22^.

The respiratory viruses and bacteria tested in this study were the most frequent microorganism detected among French pilgrims, according to previous studies^28,29^. All other pathogens (adenovirus, bocavirus, metapneumovirus, respiratory syncytial virus, parainfluenza viruses, *Bordetella pertussis, Mycoplasma pneumoniae, Neisseria meningitidis*) with prevalence <1% were not included in this study.

The acquisition of respiratory bacteria and viruses was defined as negative before travel and positive during the Hajj and/or when returning to France.

### Statistical analysis

STATA software version 11.1 (Copyright 2009 StataCorp LP, http://www.stata.com) was used for statistical analysis. Differences in the proportions were tested by Pearson’s chi-square or Fisher’s exact tests when appropriate. In order to evaluate the potential acquisition of respiratory pathogens in Saudi Arabia, we used the McNemar’s Test to compare their prevalence before leaving France and in Saudi Arabia (during and after the Hajj). Unadjusted associations between multiple factors and prevalence of respiratory pathogen acquisition and prevalence of respiratory symptom during the Hajj were examined by univariable analysis. The results were presented by percentages and risk ratio with 95% confidence interval (95%CI). Results with a p value ≤0.05 was considered statistically significant. Only the variables with a prevalence ≥5.0% were considered for statistical analysis. Variables with p values <0.2 in the univariable analysis were included in the multivariable analysis. Log-binomial regression was used to estimate factor’s adjusted risk ratios regarding respiratory symptoms and respiratory pathogens acquisition^43^.

## Supplementary information

Supplementary information
