# Supplementary material for: Respiratory tract infections among French Hajj pilgrims from 2014 to 2017
Source: Sci Rep. 2019 Nov 28;9:17771. doi: 10.1038/s41598-019-54370-0 (PMC6883043; doi:10.1038/s41598-019-54370-0)
Supplement: Supplementary file 1 — Supplementary information [file 41598_2019_54370_MOESM1_ESM.docx]

**Respiratory tract infections among French Hajj pilgrims from 2014 to 2017**

Van-Thuan Hoang^1,2,3^, Saliha Ali-Salem^1,2^, Khadidja Belhouchat^1,2^, Mohammed Meftah^1,2^, Doudou Sow^1,2,4^, Thi-Loi Dao^1,2,3^, Tran Duc Anh Ly^1,2^, Tassadit Drali^1,2^, Laetitia Ninove^5^, Saber Yezli^6^, Badriah Alotaibi^6^, Didier Raoult^2,7^, Philippe Parola^1,2^, Vincent Pommier de Santi^1,2,8^, Philippe Gautret^1,2^*

^1^Aix Marseille Univ, IRD, AP-HM, SSA, VITROME, Marseille, France

^2^IHU-Méditerranée Infection, Marseille, France

^3^Thai Binh University of Medicine and Pharmacy, Thai Binh, Viet Nam

^4^Service de Parasitologie-Mycologie, Faculté de médecine, Université Cheikh Anta Diop, Dakar, Senegal

^5^Unité des Virus Émergents (UVE: Aix-Marseille Univ – IRD 190 – Inserm 1207 – IHU Méditerranée Infection), Marseille, France

^6^The Global Centre for Mass Gatherings Medicine, Ministry of Health, Riyadh, Saudi Arabia

^7^Aix Marseille Univ, MEPHI, Marseille, France

^8^French Military Center for Epidemiology and Public Health Marseille, France

*Corresponding author:

Philippe Gautret

VITROME, Institut Hospitalo-Universitaire Méditerranée Infection, 19-21 Boulevard Jean Moulin 13385 Marseille Cedex 05, France. Phone: + 33 (0) 4 13 73 24 01. Fax: + 33 (0) 4 13 73 24 02. E-mail address: philippe.gautret@club-internet.fr

Table supplementary 1: Risk factor for respiratory symptoms during the Hajj (univariate analysis)

| **Variables** | | | | | **Cough** | | **ILI** | | **At least one symptom** | |
| --- | --- | --- | --- | --- | --- | --- | --- | --- | --- | --- |
|  |  |  |  |  | **n (%)** | **RR [95%CI]**  **p** | **n (%)** | **RR [95%CI]**  **p** | **n (%)** | **RR [95%CI]**  **p** |
| **Socio-demographic characteristics** | | | | | | | | | | |
| Gender | Male | | | | 139 (68.5) | 0.88 [0.79-0.99]  0.02 | 23 (11.3) | 0.54 [0.34-.084]  0.005 | 159 (78.3) | 0.92 [0.84-1.01] 0.07 |
|  | Female | | | | 206 (77.7) |  | 56 (21.1) |  | 225 (84.9) |  |
| Age | ≥ 60 years | | | | 206 (77.4) | 1.13 [1.01-1.27]  0.03 | 46 (17.3) | 1.08 [0.72-1.63]  0.71 | 226 (85.0) | 1.09 [0.99-1.19] 0.053 |
|  | <60 years | | | | 137 (68.5) |  | 32 (16.0) |  | 156 (78.0) |  |
| Country of birth | France | | | | 26 (65.0) | Ref | 6 (15.0) | Ref | 32 (80.0) | Ref |
|  | North Africa | | | | 307 (74.5) | 1.15 [0.91-1.45]  0.25 | 70 (17.0) | 1.13 [0.53-2.44]  0.75 | 340 (82.5) | 1.03 [0.88-1.21]  0.71 |
| **Comorbidities** | | | | | | | | | | |
| Diabetes mellitus | | | | Yes | 103 (78.0) | 1.09 [0.97-1.22]  0.17 | 25 (18.9) | 1.19 [0.78-1.84]  0.42 | 112 (84.8) | - 1. [0.96-1.15]   0.31 |
|  |  |  |  | No | 240 (71.9) |  | 53 (15.9) |  | 270 (80.8) |  |
| Hypertension | | | | Yes | 102 (74.5) | 1.02 [0.90-1.14]  0.79 | 23 (16.8) | 1.01 [0.64-1.57]  0.99 | 117 (85.4) | 1.06 [0.97-1.16]  0.21 |
|  |  |  |  | No | 241 (73.3) |  | 55 (16.7) |  | 265 (80.5) |  |
| Chronic respiratory disease | | | | Yes | 48 (87.3) | 1.22 [1.08-1.37]  0.01 | 15 (27.2) | 1.78 [1.09-2.90]  0.03 | 51 (92.7) | 1.15 [1.05-1.26]  0.03 |
|  |  |  |  | No | 295 (71.8) |  | 63 (15.3) |  | 331 (80.5) |  |
| Chronic heart disease | | | | Yes | 26 (83.9) | 1.15 [0.98-1.36]  0.18 | 7 (22.6) | 1.38 [0.70-2.74]  0.37 | 29 (93.5) | 1.15 [1.04-1.28]  0.08 |
|  |  |  |  | No | 317 (72.9) |  | 71 (16.3) |  | 353 (81.1) |  |
| Indication for vaccination against IPD | | | | Yes | 239 (77.1) | 1.16 [1.02-1.31]  0.02 | 58 (18.7) | 1.46 [0.91-2.34]  0.11 | 262 (84.5) | 1.10 [1.01-1.21]  0.04 |
|  |  |  |  | No | 104 (66.7) |  | 20 (12.8) |  | 120 (76.9) |  |
| BMI^1^ | | Normal | | | 90 (69.8) | Ref | 23 (17.8) | Ref | 104 (80.6) | Ref |
|  |  | Overweight | | | 158 (73.5) | 1.05 [0.92-1.21]  0.46 | 31 (14.4) | 0.81 [0.49-1.32]  0.40 | 175 (81.4) | 1.01 [0.91-1.12]  0.86 |
|  |  | Obesity | | | 94 (77.7) | 1.11 [0.96-1.29]  0.16 | 24 (19.8) | 1.11 [0.66-1.86]  0.69 | 102 (84.3) | 1.04 [0.93-1.17]  0.44 |
| **Preventive measures** | | | | | | | | | | |
| Vaccination against influenza | | | | Yes | 92 (72.4) | 0.98 [0.86-1.11]  0.73 | 16 (12.6) | 0.69 [0.41-1.15]  0.14 | 100 (78.7) | 0.95 [0.85-1.05]  0.27 |
|  |  |  |  | No | 251 (74.0) |  | 62 (18.3) |  | 282 (83.2) |  |
| Vaccination against IPD | | | | Yes | 70 (72.9) | 0.99 [0.86-1.13]  0.86 | 20 (20.8) | 1.33 [0.84-2.10]  0.23 | 78 (81.3) | 0.99 [0.89-1.10]  0.84 |
|  |  |  |  | No | 273 (73.8) |  | 58 (15.7) |  | 304 (82.2) |  |
| Mask | | | | Yes | 197 (75.5) | - 1. [0.94-1.18]   0.36 | 56 (21.5) | 1.91 [1.22-3.00]  0.004 | 218 (83.5) | 1.04 [0.96-1.14]  0.32 |
|  |  |  |  | No | 147 (71.7) |  | 23 (11.2) |  | 164 (80.0) |  |
| Hand washing | More often than usual | | | | 147 (75.0) | 1.03 [0.92-1.15]  0.62 | 30 (15.3) | 0.84 [0.56-1.28]  0.42 | 164 (83.7) | 1.04 [0.95-1.13]  0.42 |
|  | As usual | | | | 197 (73.0) |  | 49 (18.2) |  | 218 (80.7) |  |
| Disinfectant gel | Yes | | | | 176 (74.9) | - 1. [0.92-1.15]   0.59 | 47 (20.0) | 1.44 [0.96-2.18]  0.08 | 197 (83.8) | 1.05 [0.96-1.14]  0.29 |
|  | No | | | | 168 (72.7) |  | 32 (13.9) |  | 185 (80.1) |  |
| Disposable handkerchiefs | Yes | | | | 267 (77.8) | 1.24 [1.07-1.44]  0.001 | 61 (17.8) | 1.22 [0.75-1.97]  0.42 | 289 (84.3) | 1.11 [1.01-1.24]  0.03 |
|  | No | | | | 77 (62.6) |  | 18 (14.6) |  | 93 (75.6) |  |
| **Respiratory pathogens carrying during the Hajj** | | | | | | | | | | |
| **Respiratory virus** | | | | | | | | | | |
| At least one virus | | | Yes | | 131 (79.4) | 1.12 [1.01-1.25]  0.05 | 35 (21.2) | 1.41 [0.94-2.11]  0.09 | 147 (89.1) | 1.13 [1.04-1.23]  0.005 |
|  |  |  | No | | 203 (71.0) |  | 43 (15.0) |  | 225 (78.7) |  |
| Human rhinovirus | | | Yes | | 108 (79.4) | 1.11 [0.99-1.24]  0.09 | 28 (20.6) | 1.30 [0.86-1.97]  0.22 | 122 (89.7) | 1.13 [1.04-1.22]  0.01 |
|  | | | No | | 226 (71.7) |  | 50 (15.9) |  | 250 (79.4) |  |
| Human coronavirus | | | Yes | | 33 (86.8) | 1.19 [1.04-1.37]  0.06 | 10 (26.3) | 1.59 [0.90-2.83]  0.13 | 36 (94.7) | 1.17 [1.07-1.27]  0.03 |
|  |  |  | No | | 300 (72.8) |  | 68 (16.5) |  | 335 (81.3) |  |
| **Respiratory bacteria** | | | | | | | | | | |
| At least one bacteria | | | Yes | | 262 (74.4) | 1.02 [0.89-1.17]  0.73 | 65 (18.5) | 1.41 [0.81-2.44]  0.22 | 292 (83.0) | 1.03 [0.92-1.14]  0.62 |
|  |  |  | No | | 72 (72.7) |  | 13 (13.1) |  | 80 (80.8) |  |
| *S. aureus* | | | Yes | | 78 (73.4) | 0.99 [0.87-1.13]  0.92 | 26 (24.5) | 1.61 [1.07-2.46]  0.03 | 85 (80.2) | 0.97 [0.87-1.07]  0.49 |
|  |  |  | No | | 254 (74.1) |  | 52 (15.2) |  | 285 (83.1) |  |
| *S. pneumoniae* | | | Yes | | 70 (79.5) | 1.10 [0.97-1.24]  0.18 | 12 (13.6) | 0.76 [0.43-1.34]  0.32 | 77 (87.5) | 1.08 [0.98-1.18]  0.16 |
|  |  |  | No | | 261 (72.5) |  | 65 (18.1) |  | 292 (81.1) |  |
| *H. influenzae* | | | Yes | | 182 (76.2) | - 1. [0.95-1.19]   0.27 | 41 (17.2) | 1.01 [0.67-1.51]  0.98 | 202 (84.5) | 1.06 [0.97-1.15]  0.22 |
|  |  |  | No | | 151 (71.6) |  | 36 (17.1) |  | 169 (80.1) |  |
| *K. pneumoniae* | | | Yes | | 93 (76.2) | - 1. [0.93-1.18]   0.49 | 23 (18.9) | 1.14 [0.73-1.77]  0.57 | 104 (85.2) | 1.05 [0.96-1.15]  0.33 |
|  |  |  | No | | 238 (73.0) |  | 54 (16.6) |  | 265 (81.3) |  |
| **Bacteria co-infections** | | | | | | | | | | |
| *H. influenzae – S. pneumoniae* | | | Yes | | 42 (85.7) | 1.18 [1.04-1.34]  0.05 | 7 (14.3) | 0.81 [0.39-1.66]  0.56 | 47 (95.9) | 1.19 [1.10-1.28]  0.01 |
|  |  |  | No | | 292 (72.6) |  | 71 (17.7) |  | 325 (80.8) |  |
| *H. influenzae – K. pneumoniae* | | | Yes | | 59 (84.3) | 1.17 [1.04-1.32]  0.03 | 13 (18.6) | 1.09 [0.64-1.86]  0.76 | 63 (90.0) | 1.11 [1.01-1.22]  0.07 |
|  |  |  | No | | 275 (72.2) |  | 65 (17.1) |  | 309 (81.1) |  |
| *H. influenzae – S. aureus* | | | Yes | | 40 (75.5) | 1.02 [0.87-1.20]  0.80 | 12 (22.6) | 1.37 [0.79-2.35]  0.27 | 43 (81.1) | 0.98 [0.86-1.13]  0.78 |
|  |  |  | No | | 294 (73.9) |  | 66 (16.6) |  | 329 (82.7) |  |
| *S. pneumoniae – K. pneumoniae* | | | Yes | | 21 (84.0) | 1.14 [0.95-1.37]  0.24 | 2 (8.0) | 0.45 [0.12-1.72]  0.21 | 23 (92.0) | 1.12 [0.99-1.27]  0.20 |
|  |  |  | No | | 313 (73.5) |  | 76 (17.8) |  | 349 (81.9) |  |
| *S. pneumoniae – S. aureus* | | | Yes | | 16 (72.7) | 0.98 [0.76-1.27]  0.88 | 2 (9.1) | 0.51 [0.13-1.95]  0.30 | 17 (77.3) | 0.93 [0.74-1.18]  0.51 |
|  |  |  | No | | 318 (74.1) |  | 76 (17.7) |  | 355 (82.8) |  |
| *K. pneumoniae – S. aureus* | | | Yes | | 19 (76.0) | 1.03 [0.82-1.29]  0.82 | 7 (28.0) | 1.68 [0.87-3.26]  0.15 | 21 (84.0) | 1.02 [0.85-1.22]  0.84 |
|  |  |  | No | | 315 (73.9) |  | 71 (16.7) |  | 351 (82.4) |  |
| **Virus-bacteria co-infections** | | | | | | | | | | |
| At least one virus-bacteria combinaison | | | Yes | | 104 (80.6) | 1.13 [1.01-1.26]  0.04 | 32 (24.8) | 1.73 [1.16-2.60]  0.008 | 115 (89.1) | 1.12 [1.03-1.21]  0.02 |
|  |  |  | No | | 230 (71.4) |  | 46 (14.3) |  | 257 (79.8) |  |
| *H. influenzae* – virus | | | Yes | | 72 (82.8) | 1.15 [1.02-1.29]  0.04 | 18 (20.7) | 1.26 [0.78-2.01]  0.35 | 79 (90.8) | 1.13 [1.04-1.23]  0.02 |
|  |  |  | No | | 262 (72.0) |  | 60 (16.5) |  | 293 (80.5) |  |
| *S. pneumoniae* - virus | | | Yes | | 31 (81.6) | 1.11 [0.95-1.31]  0.27 | 8 (21.1) | 1.24 [0.65-2.38]  0.52 | 34 (89.5) | 1.09 [0.97-1.23]  0.24 |
|  |  |  | No | | 303 (73.4) |  | 70 (17.0) |  | 338 (81.8) |  |
| *K. pneumoniae -* virus | | | Yes | | 35 (83.3) | 1.14 [0.98-1.32]  0.15 | 11 (26.2) | 1.60 [0.92-2.78]  0.11 | 40 (95.2) | 1.17 [1.08-1.27]  0.02 |
|  |  |  | No | | 299 (73.1) |  | 67 (16.4) |  | 332 (81.1) |  |
| *S. aureus -* virus | | | Yes | | 36 (81.8) | 1.12 [0.96-1.30]  0.21 | 12 (27.3) | 1.68 [0.99-2.86]  0.07 | 40 (90.9) | 1.11 [1.00-1.24]  0.12 |
|  |  |  | No | | 298 (73.2) |  | 66 (16.2) |  | 332 (81.6) |  |

*IPD: Invasive pneumococcal disease, ILI: influenza-like illness*

^1^Normal weight: BMI: 18.5 – 24.9, Overweight: BMI 25.0 – 29.9, Obesity: BMI ≥30

Table supplementary 2: Risk factor for acquisition of respiratory viruses during the Hajj (univariate analysis)

| **Variables** | | | | | **Influenza viruses** | | **Human rhinovirus** | | **Human coronaviruses** | |
| --- | --- | --- | --- | --- | --- | --- | --- | --- | --- | --- |
|  |  |  |  |  | **n (%)** | **RR [95%CI] p** | **n (%)** | **RR [95%CI] p** | **n (%)** | **RR [95%CI] p** |
| **Socio-demographic characteristics** | | | | | | | | | | |
| Gender | | | Male | | 4 (2.0) | 0.43 [0.14-1.32]  0.13 | 51 (26.4) | 0.97 [0.71-1.32]  0.84 | 10 (5.1) | 0.50 [0.25-1.01]  0.05 |
|  |  |  | Female | | 12 (4.7) |  | 69 (27.3) |  | 26 (10.2) |  |
| Age | | | 60 years | | 13 (5.1) | 3.23 [0.93-11.19]  0.05 | 70 (27.6) | 1.04 [0.76-1.42]  0.80 | 29 (11.3) | 3.06 [1.37-6.84]  0.004 |
|  |  |  | 60 years | | 3 (1.6) |  | 50 (26.5) |  | 7 (3.7) |  |
| Country of birth | | | France | | 2 (5.3) | Ref | 8 (21.1) | Ref | 1 (2.6) | Ref |
|  |  |  | North Africa | | 13 (3.3) | 0.63 [0.15-2.67]  0.75 | 110 (28.1) | 1.33 [0.71-2.52]  0.38 | 35 (8.8) | 3.36 [0.47-23.84]  0.23 |
| **Comorbidities** | | | | | | | | | | |
| Diabetes mellitus | | | | Yes | 4 (3.1) | 0.84 [0.28-2.55]  0.75 | 35 (28.0) | 1.04 [0.75-1.46]  0.80 | 11 (8.7) | - 1. [0.56-2.18]   0.77 |
|  |  |  |  | No | 12 (3.8) |  | 85 (26.8) |  | 25 (7.8) |  |
| Hypertension | | | | Yes | 3 (2.3) | 0.56 [0.16-1.94]  0.35 | 42 (32.8) | 1.32 [0.97-1.81]  0.09 | 12 (9.2) | 1.20 [0.62-2.33]  0.59 |
|  |  |  |  | No | 13 (4.1) |  | 78 (24.8) |  | 24 (7.6) |  |
| Chronic respiratory disease | | | | Yes | 3 (5.6) | 1.68 [0.49-5.69]  0.41 | 17 (32.1) | 1.21 [0.79-1.85]  0.39 | 5 (9.3) | 1.17 [0.48-2.88]  0.73 |
|  |  |  |  | No | 13 (3.3) |  | 103 (26.5) |  | 31 (7.9) |  |
| Chronic heart disease | | | | Yes | 1 (3.2) | 0.89 [0.12-6.54]  0.91 | 10 (33.3) | 1.25 [0.73-2.12]  0.43 | 3 (9.7) | 1.21 [0.40-3.75]  0.73 |
|  |  |  |  | No | 15 (3.6) |  | 110 (26.7) |  | 33 (7.9) |  |
| Indication for vaccination against IPD | | | | Yes | 13 (4.4) | 2.15 [0.62-7.44]  0.21 | 80 (27.2) | - 1. [0.73-1.39]   0.97 | 30 (10.0) | 2.46 [1.05-5.77]  0.03 |
|  |  |  |  | No | 3 (2.0) |  | 40 (27.0) |  | 6 (4.1) |  |
| BMI^1^ | | Normal | | | 5 (4.1) | Ref | 30 (24.6) | Ref | 7 (5.7) | Ref |
|  |  | Overweight | | | 8 (3.9) | 0.95 [0.32-2.83]  0.92 | 56 (26.7) | 1.09 [0.74-1.59]  0.68 | 17 (8.2) | 1.42 [0.61-3.34]  0.42 |
|  |  | Obesity | | | 3 (2.6) | 0.64 [0.16-2.60]  0.53 | 35 (30.4) | 1.24 [0.82-1.88]  0.32 | 12 (10.2) | 1.77 [0.72-4.35]  0.21 |
| **Preventive measures** | | | | | | | | | | |
| Vaccination against influenza | | | | Yes | 5 (4.0) | 1.18 [0.42-3.33]  0.75 | 37 (30.3) | 1.17 [0.84-1.62]  0.35 | 10 (8.1) | 0.99 [0.50-2.01]  0.99 |
|  |  |  |  | No | 11 (3.4) |  | 83 (25.9) |  | 26 (8.1) |  |
| Vaccination against IPD | | | | Yes | 2 (2.1) | 0.53 [0.12-2.31]  0.39 | 27 (28.7) | 1.07 [0.75-1.54]  0.70 | 5 (5.3) | 0.60 [0.24-1.51]  0.27 |
|  |  |  |  | No | 14 (4.0) |  | 93 (26.7) |  | 31 (8.8) |  |
| Mask | | | | Yes | 9 (3.5) | 0.98 [0.37-2.58]  0.96 | 79 (40.0) | 1.42 [1.02-1.97]  0.03 | 21 (8.3) | 1.06 [0.56-2.01]  0.85 |
|  |  |  |  | No | 7 (3.6) |  | 41 (21.8) |  | 15 (7.8) |  |
| Hand washing | More often than usual | | | | 5 (2.6) | 0.62 [0.22-1.76]  0.36 | 51 (27.3) | - 1. [0.74-1.38]   0.94 | 14 (7.4) | 0.87 [0.46-1.65]  0.67 |
|  | As usual | | | | 11 (4.2) |  | 69 (27.0) |  | 22 (8.5) |  |
| Disinfectant gel | | | | Yes | 8 (3.5) | 0.98 [0.37-2.56]  0.96 | 60 (26.8) | 0.98 [0.72-1.33]  0.88 | 16 (7.1) | 0.78 [0.42-1.47]  0.44 |
|  |  |  |  | No | 8 (3.6) |  | 60 (27.4) |  | 20 (9.0) |  |
| Disposable handkerchiefs | | | | Yes | 13 (4.0) | 1.55 [0.45-5.36]  0.48 | 89 (27.5) | 1.05 [0.74-1.50]  0.77 | 27 (8.2) | 1.08 [0.52-2.22]  0.84 |
|  |  |  |  | No | 3 (2.5) |  | 31 (26.1) |  | 9 (7.6) |  |

^1^BMI normal: 18.5 – 24.9, Overweight: BMI 25.0 – 29.9, Obesity: BMI ≥30

Table supplementary 3: Risk factor for acquisition of respiratory bacteria during the Hajj (univariate analysis)

| **Variables** | | | | ***S. aureus*** | | ***S. pneumoniae*** | | ***H. influenzae*** | | ***K. pneumoniae*** | |
| --- | --- | --- | --- | --- | --- | --- | --- | --- | --- | --- | --- |
|  |  |  |  | **n (%)** | **RR (95%CI) p** | **n (%)** | **RR (95%CI) p** | **n (%)** | **RR (95%CI) p** | **n (%)** | **RR (95%CI) p** |
| **Socio-demographic characteristics** | | | | | | | | | | | |
| Gender | | Male | | 29 (14.8) | 1.11 [0.70-1.75]  0.67 | 39 (20.0) | 1.22 [0.82-1.82]  0.32 | 61 (31.6) | 0.99 [0.76-1.31]  0.99 | 32 (16.3) | 0.67 [0.46-0.98]  0.04 |
|  |  | Female | | 34 (13.4) |  | 41 (16.3) |  | 83 (31.7) |  | 62 (24.4) |  |
| Age | | 60 years | | 34 (13.2) | 0.87 [0.55-1.37]  0.54 | 51 (20.2) | 1.32 [0.87-2.00]  0.19 | 90 (35.0) | 1.30 [0.97-1.72]  0.07 | 54 (21.2) | 1.07 [0.74-1.56]  0.70 |
|  |  | 60 years | | 29 (15.3) |  | 29 (15.3) |  | 53 (27.4) |  | 38 (19.8) |  |
| Country of birth | | France | | 8 (21.1) | Ref | 4 (10.8) | Ref | 10 (25.6) | Ref | 8 (20.5) | Ref |
|  |  | North Africa | | 52 (13.1) | 0.62 [0.62-1.21  0.17] | 73 (18.6) | 1.72 [0.67-4.45]  0.26 | 129 (32.3) | 1.26 [0.72-2.19]  0.42 | 82 (20.9) | - 1. [0.53-1.94]   0.96 |
| **Comorbidities** | | | | | | | | | | | |
| Diabetes mellitus | | | Yes | 19 (14.8) | 1.07 [0.65-1.76]  0.78 | 19 (15.1) | 0.78 [0.49-1.25]  0.30 | 44 (33.8) | 1.10 [0.83-1.48]  0.51 | 27 (21.6) | 1.06 [0.71-1.58]  0.76 |
|  |  |  | No | 44 (13.8) |  | 61 (19.3) |  | 99 (30.7) |  | 65 (20.3) |  |
| Hypertension | | | Yes | 16 (12.3) | 0.83 [0.49-1.40]  0.48 | 25 (19.4) | 1.10 [0.72-1.69]  0.65 | 41 (30.8) | 0.97 [0.72-1.31]  0.83 | 32 (24.8) | 1.31 [0.90-1.90]  0.17 |
|  |  |  | No | 47 (14.9) |  | 55 (17.6) |  | 102 (31.9) |  | 60 (18.9) |  |
| Chronic respiratory disease | | | Yes | 8 (15.1) | 1.08 [0.54-2.14]  0.83 | 10 (18.5) | - 1. [0.56-1.87]   0.93 | 27 (48.2) | 1.65 [1.21-2.25]  0.004 | 11 (20.0) | 0.96 [0.55-1.69]  0.90 |
|  |  |  | No | 55 (14.0) |  | 70 (18.0) |  | 116 (29.2) |  | 81 (20.8) |  |
| Chronic heart disease | | | Yes | 2 (0.44) | 0.44 [0.11-1.71]  0.21 | 7 (22.6) | 1.27 [0.64-2.52]  0.50 | 9 (29.0) | 0.91 [0.52-1.61]  0.75 | 9 (29.0) | 1.45 [0.81-2.59]  0.23 |
|  |  |  | No | 61 (14.7) |  | 73 (17.8) |  | 134 (31.8) |  | 83 (20.0) |  |
| Indication for vaccination against IPD | | | Yes | 40 (13.4) | 0.86 [0.54-1.39]  0.55 | 54 (18.3) | - 1. [0.68-1.58]   0.87 | 98 (32.6) | 1.10 [0.82-1.48]  0.52 | 61 (20.6) | 0.99 [0.67-1.46]  0.96 |
|  |  |  | No | 23 (15.5) |  | 26 (17.7) |  | 45 (29.6) |  | 31 (20.8) |  |
| BMI^1^ | Normal | | | 15 (12.3) | Ref | 23 (18.9) | Ref | 45 (36.6) | Ref | 28 (22.8) | Ref |
|  | Overweight | | | 32 (15.3) | 1.25 [0.70-2.20]  0.45 | 40 (19.2) | - 1. [0.64-1.62]   0.93 | 64 (30.2) | 0.83 [0.61-1.12]  0.22 | 41 (19.7) | 0.87 [0.57-1.33]  0.51 |
|  | Obesity | | | 16 (13.8) | 1.12 [0.58-2.16]  0.73 | 17 (15.0) | 0.80 [0.45-1.41]  0.44 | 35 (29.9) | 0.82 [0.57-1.17]  0.28 | 25 (21.6) | 0.95 [0.59-1.52]  0.82 |
| **Preventive measures** | | | | | | | | | | | |
| Vaccination against influenza | | | Yes | 19 (15.3) | 1.12 [0.68-1.84]  0.65 | 24 (19.5) | 1.11 [0.72-1.71]  0.63 | 36 (28.8) | 0.88 [0.64-1.21]  0.43 | 24 (19.5) | 0.92 [0.61-1.40]  0.71 |
|  |  |  | No | 44 (13.7) |  | 56 (17.6) |  | 107 (32.6) |  | 68 (21.1) |  |
| Vaccination against IPD | | | Yes | 13 (13.8) | 0.97 [0.55-1.72]  0.93 | 6 (6.4) | 0.30 [0.13-0.67]  0.001 | 38 (40.0) | 1.36 [1.02-1.83]  0.05 | 25 (26.6) | 1.39 [0.93-2.08]  0.11 |
|  |  |  | No | 50 (14.2) |  | 74 (21.3) |  | 105 (29.3) |  | 67 (19.1) |  |
| Mask | | | Yes | 39 (15.4) | 1.23 [0.77-1.97]  0.39 | 39 (15.4) | 0.71 [0.48-1.06]  0.10 | 88 (34.6) | 1.19 [0.90-1.58]  0.21 | 48 (19.0) | 0.81 [0.56-1.16]  0.25 |
|  |  |  | No | 24 (12.5) |  | 41 (21.6) |  | 56 (29.0) |  | 45 (23.4) |  |
| Hand washing | | More often than usual | | 22 (11.6) | 0.73 [0.45-1.18]  0.20 | 38 (20.1) | 1.22 [0.82-1.81]  0.33 | 67 (35.6) | 1.20 [0.92-1.57]  0.19 | 34 (18.1) | 0.79 [0.54-1.15]  0.21 |
|  |  | As usual | | 41 (16.0) |  | 42 (16.5) |  | 77 (29.7) |  | 59 (23.0) |  |
| Disinfectant gel | | | Yes | 31 (13.7) | 0.94 [0.60-1.49]  0.80 | 44 (19.8) | 1.22 [0.82-1.81]  0.33 | 65 (29.1) | 0.83 [0.63-1.08]  0.17 | 50 (22.3) | 1.15 [0.80-1.65]  0.46 |
|  |  |  | No | 32 (14.5) |  | 36 (16.3) |  | 79 (35.3) |  | 43 (19.5) |  |
| Disposable handkerchiefs | | | Yes | 39 (11.9) | 0.58 [0.37-0.93]  0.02 | 67 (20.6) | 1.85 [1.06-3.22]  0.02 | 100 (30.2) | 0.80 [0.60-1.06]  0.13 | 72 (21.9) | 1.21 [0.78-1.87]  0.39 |
|  |  |  | No | 24 (20.3) |  | 13 (11.1) |  | 44 (37.9) |  | 21 (18.1) |  |
| Antibiotic use | | | Yes | 25 (14.0) | 0.99 [0.62-1.57]  0.95 | 25 (14.1) | 0.69 [0.44-1.06]  0.08 | 60 (33.5) | 1.08 [0.82-1.41]  0.59 | 44 (24.7) | 1.32 [0.93-1.90]  0.12 |
|  |  |  | No | 38 (14.2) |  | 55 (20.6) |  | 84 (31.1) |  | 50 (18.7) |  |

^1^BMI normal: 18.5 – 24.9, Overweight: BMI 25.0 – 29.9, Obesity: BMI ≥30
